# TLR2 Deficiency Exacerbates Imiquimod-Induced Psoriasis-Like Skin Inflammation through Decrease in Regulatory T Cells and Impaired IL-10 Production

**DOI:** 10.3390/ijms21228560

**Published:** 2020-11-13

**Authors:** Momoko Nakao, Makoto Sugaya, Hideki Fujita, Tomomitsu Miyagaki, Sohshi Morimura, Sayaka Shibata, Yoshihide Asano, Shinichi Sato

**Affiliations:** 1 Department of Dermatology, The University of Tokyo Graduate School of Medicine, Tokyo 113-8655, Japan; orinomomoco@gmail.com (M.N.); hfuji-tky@umin.ac.jp (H.F.); asahikari1979@gmail.com (T.M.); sohshimorimura@gmail.com (S.M.); SHIBATAS-DER@h.u-tokyo.ac.jp (S.S.); asanoyoshihide@hotmail.com (Y.A.); satos-der@h.u-tokyo.ac.jp (S.S.); 2 Department of Dermatology, International University of Health and Welfare, Chiba 286-8520, Japan; 3 Division of Cutaneous Science, Department of Dermatology, Nihon University School of Medicine, Tokyo 173-8610, Japan; 4 Department of Dermatology, St. Marianna University School of Medicine, Kanagawa 216-8511, Japan

**Keywords:** psoriasis, TLR2, imiquimod, regulatory T cells, dendritic cells

## Abstract

Emerging evidence has demonstrated that Toll-like receptors (TLRs) are associated with autoimmune diseases. In this study, we investigated the role of TLR2 in psoriasis using imiquimod-induced psoriasis-like dermatitis. Although TLR2 signaling is known to play a critical role in the induction of proinflammatory cytokines by immune cells, such as dendritic cells (DCs), macrophages, and monocytes, TLR2 deficiency unexpectedly exacerbated psoriasiform skin inflammation. Importantly, messenger RNA (mRNA) levels of Foxp-3 and IL-10 in the lesional skin were significantly decreased in TLR2 KO mice compared with wild-type mice. Furthermore, flow cytometric analysis of the lymph nodes revealed that the frequency of regulatory T cells (Tregs) among CD4-positive cells was decreased. Notably, stimulation with Pam3CSK4 (TLR2/1 ligand) or Pam2CSK4 (TLR2/6 ligand) increased IL-10 production from Tregs and DCs and the proliferation of Tregs. Finally, adoptive transfer of Tregs from wild-type mice reduced imiquimod-induced skin inflammation in TLR2 KO mice. Taken together, our results suggest that TLR2 signaling directly enhances Treg proliferation and IL-10 production by Tregs and DCs, suppressing imiquimod-induced psoriasis-like skin inflammation. Enhancement of TLR2 signaling may be a new therapeutic strategy for psoriasis.

## 1. Introduction

Psoriasis is characterized by acanthosis with massive infiltration of dendritic cells (DCs), T cells, neutrophils and macrophages [1,2]. It is widely accepted that the pathophysiology of psoriasis is related to the activation and dysregulation of the innate and adaptive immune system [2]. Environmental factors activate the innate immune system, which contributes to the initiation and maintenance of autoimmune diseases such as psoriasis [3]. Plasmacytoid DCs, activated through complexes of the antimicrobial peptide LL-37 and DNA in a toll-like receptor (TLR) 9-dependent manner, induce a proinflammatory cytokine cascade [4]. Interferon-alpha expressed by activated plasmacytoid DCs is important in the early phase of psoriasis, further activating dermal DCs and triggering downstream T cell-mediated adaptive immunity [5]. Activated DCs produce various cytokines such as IL-12 and IL-23, which are critical for differentiation and expansion of Type 1 and Type 17 T-helper cells, respectively [6]. Recently, the role of the IL-23/Th17 axis has been verified by the remarkable efficacy of human monoclonal antibodies against IL-12/23p40, IL-17, and its receptor [7]. Although many clinical studies have proved important roles of the IL-23/Th17 pathway in psoriasis [8], the pathogenesis of psoriasis has not yet been fully elucidated.

TLRs function as important sensors of both innate and adaptive immune systems [9]. Activation of TLRs induces inflammatory cytokines and chemokines, activating intracellular signaling pathways to regulate the host’s inflammatory response [10]. TLRs also recognize endogeneous host molecules and trigger inflammatory responses, and inappropriate TLR responses may cause acute and chronic inflammations as well as systemic autoimmune diseases [11]. Therefore, the roles of TLRs in autoimmune diseases are attracting attention from many researchers. TLR2 is a member of the TLR family expressed on the surface of various types of cells, such as DCs, macrophages, and lymphocytes [12,13,14]. TLR2 forms a heterodimer with TLR1 or TLR6 and responds to lipopeptides from a wide variety of microbes, including Gram-positive bacteria [15]. Previous reports showed that TLR2 was increased on peripheral blood monocytes from patients with psoriatic arthritis [16] and that the single nucleotide polymorphism rs3804099 of TLR2 has significant effects on the heritability of psoriasis vulgaris in Chinese people [17]. Furthermore, the critical role of TLR2 has been reported in other autoimmune diseases, such as experimental autoimmune encephalomyelitis and multiple sclerosis, in which IL-17 plays an important role [18,19]. The role of TLR2 in psoriasis, however, remains to be elucidated.

In this study, we investigated the possible roles of TLR2 in the development of psoriasis, using a mouse psoriasis model induced by imiquimod, a ligand for TLR7 [20]. We show here that TLR2 deficiency unexpectedly exacerbates psoriasis-like skin inflammation through a decrease in regulatory T cells (Tregs) and impaired IL-10 production by Tregs and DCs.

## 2. Results

### 2.1. TLR2 Deficiency Exacerbates Psoriasiform Dermatitis Induced by Imiquimod Treatment

**Figure 1:**
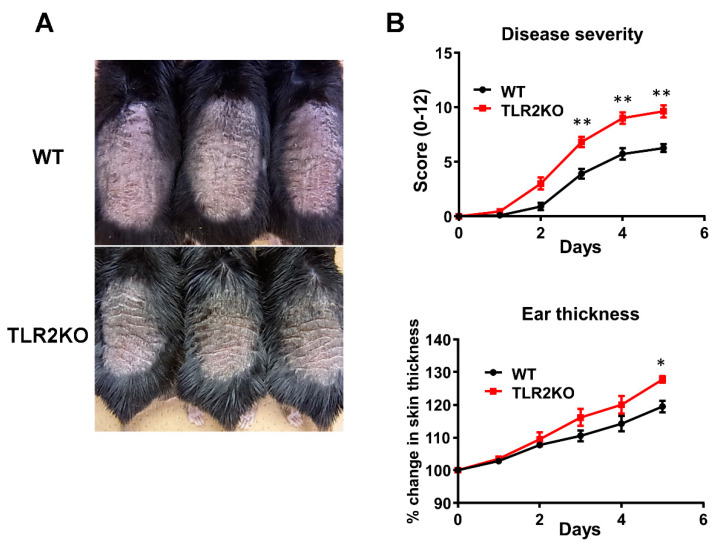
TLR2 deficiency exacerbates psoriasiform dermatitis induced by imiquimod treatment. Shaved back skin and ears of wild-type (WT) and TLR2 knockout (TLR2 KO) mice were topically treated with imiquimod for 5 consecutive days. (**A**) Phenotypical manifestation of WT and TLR2 KO mouse back skin induced by imiquimod application at day 5. (**B**) Disease severity and ear thickness during imiquimod treatment. ** *p* < 0.01. Clinical scores for disease severity were calculated daily using a scoring system based on the clinical psoriasis area and severity index. Data are presented as mean ± SEM of three independent experiments (*n* = 9 for each group). * *p* < 0.05 versus WT mice with imiquimod application.

**Figure 2:**
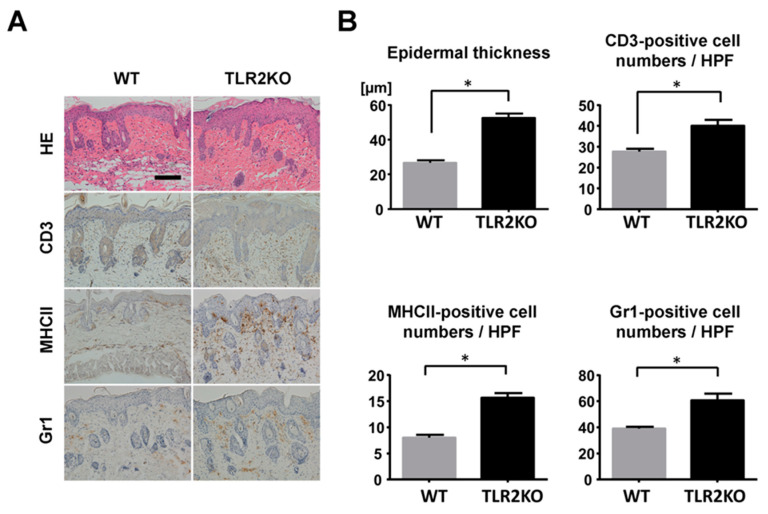
Epidermal hyperplasia and inflammatory cell infiltration are enhanced in TLR2-deficient mice. (**A**) Histological presentation of wild-type (WT) and TLR2 knockout (TLR2 KO) mouse back skin induced by imiquimod application at day 5 (×200). The sections were stained with hematoxylin and eosin (HE) or immunostained for CD3, major histocompatibility complex (MHC) class II (MHCII), or Gr1. Representative pictures from 9 mice per group. Scale bar, 50 mm. (**B**) The number of epidermal cell layers and dermal inflammatory cells were counted per high-power field. Data are presented as mean ± SEM of three independent experiments (*n* = 9; * *p* < 0.05).

To examine the role of TLR2 during psoriasiform skin inflammation, we first applied imiquimod cream on mouse back skin and ear, and investigated the clinical and histopathological features of WT and TLR2 KO mice. TLR2 KO mice exhibited worse clinical outcomes with more severe scales and thicker skin by imiquimod application than WT mice (Figure 1A). Significant differences in disease severity and ear thickness were observed between WT and TLR2 KO mice from day 3 to 5 (Figure 1B). Consistently, histological analysis of skin samples at day 5 of imiquimod treatment showed more severe epidermal hyperplasia and more intense inflammatory cell infiltration in TLR2 KO mice compared with WT mice (Figure 2A,B). CD3, major histocompatibility complex class II-positive or Gr-1-positive cells in the upper dermis were significantly increased in TLR2 KO mice compared with WT mice (Figure 2A,B). Thus, TLR2 deficiency exacerbated imiquimod-induced psoriasiform dermatitis.

### 2.2. TLR2 Deficiency Increases Th1 Cytokine mRNA Levels and Decreases Foxp3 and IL-10 mRNA Levels

**Figure 3:**
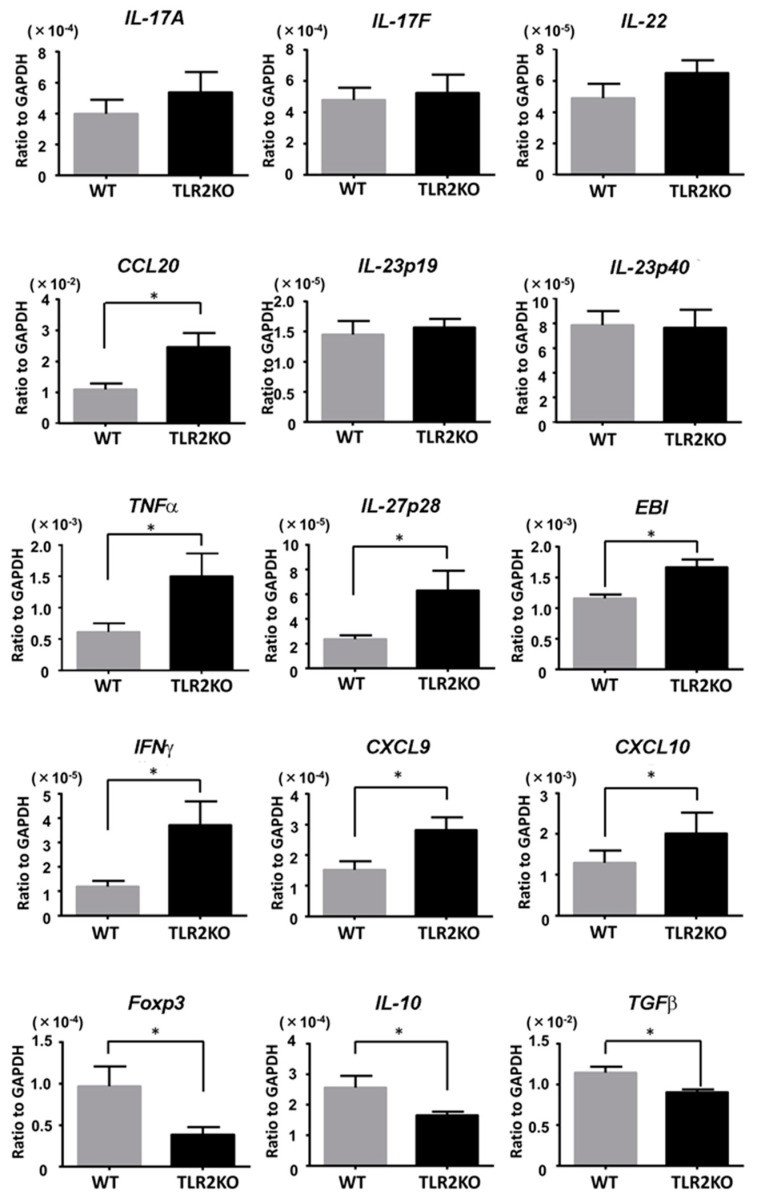
TLR2 deficiency increases Th1 cytokine mRNA levels and decreases Foxp3 and IL-10 levels. Wild-type (WT) and TLR2 knockout (TLR2 KO) mice were applied with imiquimod, and skin samples were taken 48 h after imiquimod application. Messenger RNA levels of the indicated cytokines and Foxp-3 were determined by quantitative RT-PCR. Data are obtained from duplicate samples from 12 mice in each group. Values are presented as mean ± SEM of three independent experiments. * *p* < 0.05.

We next examined the mRNA levels of psoriasis-related cytokines in the imiquimod-induced psoriasiform skin lesions in WT and TLR2 KO mice. Samples were taken from mouse back skin 48 h after imiquimod application. Although mRNA levels for *CCL20* were significantly increased in TLR2 KO mice, there was no significant difference in mRNA levels for *IL-23* (*p19*, *p40*) and other Th17 cytokines, such as *IL-17A*, *IL-17F,* and *IL-22* (Figure 3). On the contrary, mRNA levels for *TNFα* and Th1 cytokines, such as *IL-27p28*, *IL-27EBI3*, *IFNγ*, *CXCL9*, and *CXCL10*, were significantly higher in TLR2 KO mice than in WT mice (Figure 3). Importantly, mRNA levels of *Foxp3*, *IL-10*, and *TGFβ* were significantly decreased in TLR2 KO mice compared with WT mice (Figure 3), suggesting that downregulation of Tregs and impaired IL-10 production in TLR2 KO mice increased Th1 cytokine mRNA levels and exacerbated the imiquimod-induced skin inflammation.

### 2.3. TLR2 Deficiency Negatively Regulates Frequency of Tregs and IL-10 Expression in the Lymph Nodes

**Figure 4:**
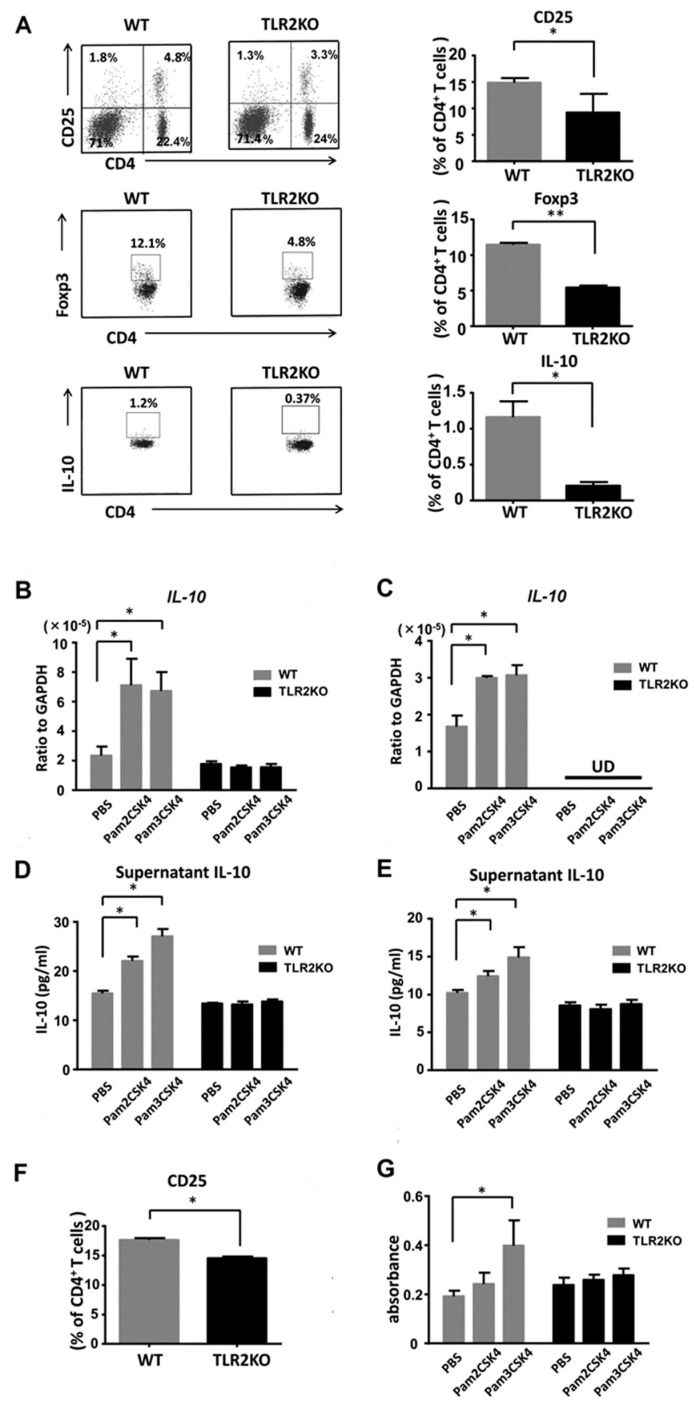
TLR2 regulates proliferation of Tregs and IL-10 production by DCs and Tregs. Wild-type (WT) and TLR2 knockout (TLR2 KO) mice were treated as described. At day 2, cell suspensions from the draining lymph nodes were stimulated with PMA and ionomycin and were stained with antibodies specific for Foxp3 and IL-10 as well as CD4 and CD25 cell surface markers, followed by flow cytometric analysis. Flow plots show the CD25-positive, Foxp3-positive, or IL-10-positive population of the lymph nodes from WT and TLR2 KO mice. Flow plots were gated on CD4-positive cells (**A**). Data are representative of three independent experiments, where each group contains three mice. The right graphs show the frequency among CD4 cells in each group. Values are presented as mean ± SEM (*n* = 9). * *p* < 0.05, ** *p* < 0.01. IL-10 mRNA expression levels by DCs (**B**) and Tregs (**C**) stimulated with either Pam2CSK4 (TLR2/6 ligand) or Pam3CSK4 (TLR2/1 ligand). IL-10 concentrations in culture supernatants of DCs (**D**) and Tregs (**E**) stimulated with Pam2CSK4 or Pam3CSK4. (**F**) The frequency of CD25-positive cells among the CD4-positive cells analyzed by flow cytometry using skin draining lymph nodes from naïve TLR2 knockout (TLR2 KO) and wild-type (WT) mice. Data are representative of three independent experiments, where each group contains two mice. (**G**) BrdU cell proliferation analysis of Tregs from TLR2 KO and WT mice. Purified Tregs were cultured for 3 days with/without stimulation of Pam3CSK4 or Pam2CSK4. Values are presented as mean ± SEM of three independent experiments in triplicates. * *p* < 0.05, UD; undetectable.

To clarify whether TLR2 deficiency directly downregulates Tregs, we next performed flow cytometric analysis of regional lymph node cells 48 h after imiquimod application. TLR2 deficiency significantly decreased the frequency of CD4 and CD25 double-positive Tregs in the draining lymph nodes after imiquimod application (Figure 4A). The absolute numbers of Tregs on average were 7.2 × 10^5^ and 5.0 × 10^5^ in WT mice and TLR2 KO mice, respectively. The proportion of Foxp3 or IL-10-positive cells among the CD4-positive cells was significantly lower in TLR2 KO mice than that of WT mice (Figure 4A), which was compatible with previous reports [21,22]. Thus, TLR2 deficiency negatively regulated the frequency of Tregs and IL-10 expression in the lymph nodes after treatment with imiquimod.

### 2.4. IL-10 Production from DCs and Tregs Is Increased by Stimulation with Either Pam2CSK4 or Pam3CSK4

We further examined IL-10 production from DCs and Tregs. We stimulated DCs and Tregs with Pam2CSK4 (TLR2/6 ligand) or Pam3CSK4 (TLR2/1 ligand) and measured *IL-10* mRNA expression levels by quantitative reverse-transcription PCR. *IL-10* mRNA expression levels were increased with stimulation of either Pam2CSK4 or Pam3CSK4 (Figure 4B,C). Next, we further analyzed IL-10 production in cell culture supernatants by ELISA. Consistently, IL-10 concentrations in the supernatants of Tregs and DCs were elevated with stimulation of either Pam2CSK4 or Pam3CSK4 (Figure 4D,E). Thus, IL-10 production from DCs and Tregs was enhanced by stimulation with either Pam2CSK4 or Pam3CSK4.

### 2.5. Pam3CSK4 Enhances the Proliferation of Tregs

To confirm the role of TLR2 in the proliferation of Tregs, we selectively stimulated TLR2 on Tregs with Pam2CSK4 or Pam3CSK4. First, we investigated the frequency of CD25-positive Tregs among CD4-positive cells in naïve mice. Flow cytometric analysis of the draining lymph nodes revealed that the proportion of CD25-positive cells among the CD4-positive cells was significantly lower in TLR2 KO mice than that of WT mice (Figure 4F). The absolute numbers of Tregs on average were 3.5 × 10^5^ and 2.0 × 10^5^ in WT mice and TLR2 KO mice, respectively. Pam3CSK4 significantly enhanced the uptake of BrdU in purified Tregs in vitro (Figure 4G). Pam2CSK4 also tended to increase the uptake, which was not statistically significant.

### 2.6. Pam3CSK4 and Pam2CSK4 Attenuate Imiquimod-Induced Skin Inflammation

**Figure 5:**
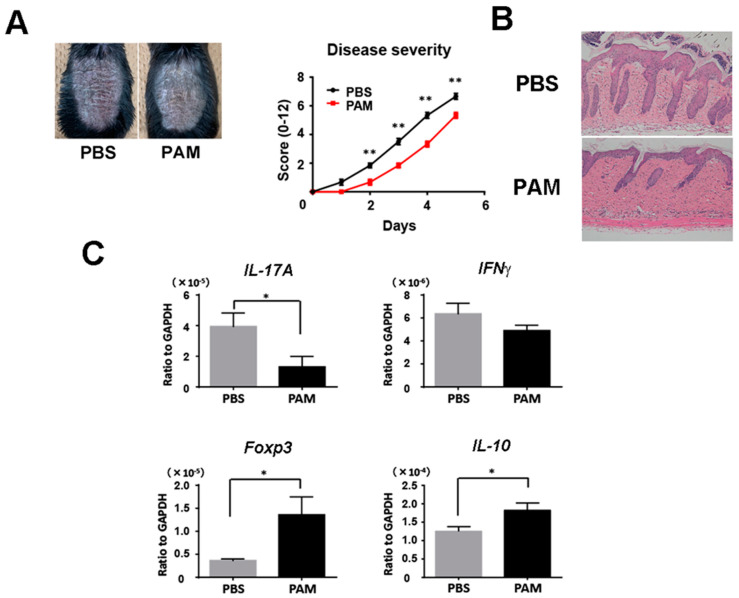
Systemic treatment with Pam3CSK4 and Pam2CSK4 attenuates imiquimod-induced skin inflammation. Shaved back skin of wild-type (WT) mice were topically treated with imiquimod for 5 consecutive days. Wild-type mice were injected with 50 g Pam3CSK4 and 50 g Pam2CSK4 (PAM) or PBS intravenously two days before, on the same day, and two days after imiquimod application. (**A**) The phenotypical manifestation of back skin induced by imiquimod application at day 5. (**B**) Disease severity during imiquimod treatment. Clinical scores for disease severity were calculated daily using a scoring system based on the clinical psoriasis area and severity index. Data are presented as mean ± SEM of three independent experiments (*n* = 9 for each group). * *p* < 0.05 versus WT mice with imiquimod application. (**C**) Messenger RNA levels of the indicated cytokines and Foxp-3 were determined by quantitative RT-PCR. Values are presented as mean ± SEM (*n* = 9). * *p* < 0.05, ** *p* < 0.01.

We next injected Pam3CSK4 and Pam2CSK4, or PBS intravenously into wild-type mice during imiquimod treatment. Psoriasiform skin inflammation was significantly decreased clinically and histologically with the injection of Pam3CSK4 and Pam2CSK4 (Figure 5A,B). Consistently, Pam3CSK4 and Pam2CSK4 significantly reduced *IL-17A* mRNA expression and upregulated *Foxp3* and *IL-10* mRNA expression in the skin lesion (Figure 5C). These results demonstrated that the enhancement of TLR2 signaling increased Tregs and improved imiquimod-induced skin inflammation.

### 2.7. Adoptive Transfer of Tregs from WT Mice Ameliorates Imiquimod-Induced Psoriasiform Dermatitis in TLR2 KO Mice

**Figure 6:**
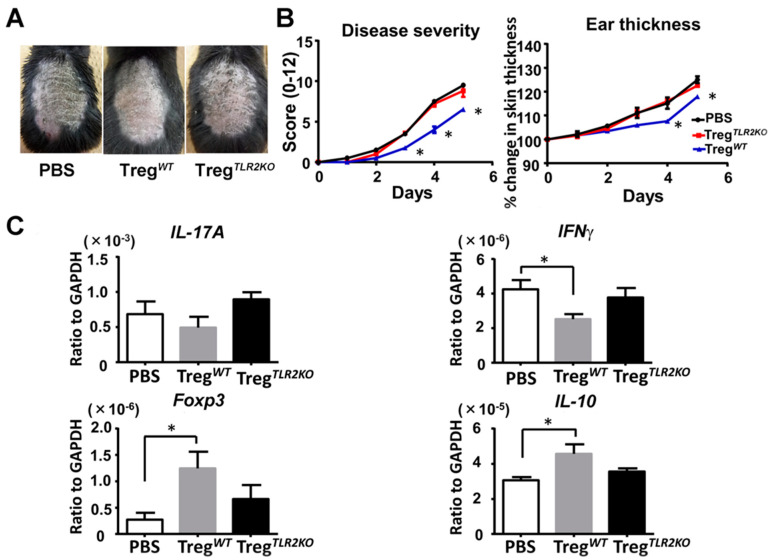
Adoptive transfer of Tregs from wild-type mice ameliorates imiquimod-induced psoriasiform dermatitis in TLR2-deficient mice. Shaved back skin and ears of TLR2 knockout (TLR2 KO) mice that had received Tregs from wild-type (WT) or TLR2 KO mice were topically treated with imiquimod for 5 consecutive days. (**A**) Phenotypical manifestation induced by imiquimod inTLR2 KO mouse that had received Tregs from WT mice, TLR2 KO mice, or PBS alone. (**B**) Disease severity and ear thickness during imiquimod treatment. Clinical scores for disease severity were calculated daily using a scoring system based on the clinical Psoriasis Area and Severity Index. Data are presented as mean ± SEM (*n* = 6 for each group). * *p* < 0.05 versus PBS treatment group. (**C**) Skin samples were taken 48h after imiquimod application. Messenger RNA levels of IL-17A, IFNγ, Foxp3, and IL-10 were determined by quantitative RT-PCR. Data were obtained from duplicate samples from 6 mice in each group. Values are presented as mean ± SEM of three independent experiments. * *p* < 0.05.

Finally, we intravenously transferred Tregs from WT mice or TLR2 KO mice into TLR2 KO mice to confirm that Tregs caused a difference in imiquimod-induced psoriasiform dermatitis. Adoptive transfer of Tregs from WT mice, but not from TLR2 KO mice, significantly attenuated skin inflammation after imiquimod treatment compared to PBS-injected TLR2KO mice (Figure 6A,B). Furthermore, the adoptive transfer of Tregs from WT mice downregulated *IFNγ* mRNA expression, accompanied by an increase in *Foxp3* and *IL-10* mRNA expression in the lesional skin (Figure 6C). Thus, the adoptive transfer of Tregs from WT mice ameliorates imiquimod-induced psoriasiform dermatitis in TLR2 KO mice.

## 3. Discussion

This study demonstrated whether and how TLR2 was involved in the pathogenesis of psoriasis using an imiquimod-induced psoriasiform dermatitis [23]. Although TLR2 signaling has been known to induce inflammatory cytokines and immune responses, TLR2 deficiency unexpectedly exacerbated psoriasiform skin inflammation. Whereas Th17 cytokines, such as *IL-17A*, *IL-17F,* and *IL-22*, were not changed, *TNFα* and Th1 cytokines such as *IFNγ*, *CXCL9*, *CXCL10*, *IL-27p28*, and *IL-27EBI3* were significantly higher in TLR2 KO mice than in WT mice. Importantly, *Foxp3* and *IL-10* expression in the lesional skin was decreased in TLR2 KO mice, leading us to focus on Tregs and IL-10.

The development and function of Tregs have been minutely investigated. Naturally occurring Tregs, the majority of which are developed in the thymus and characterized as constitutively expressing the transcription factor *Foxp3*, are essential for the maintenance of self-tolerance and immune homeostasis [24]. In addition to thymus-derived natural Tregs, conventional T cells can also acquire Treg phenotype and function in the periphery [25]. Such inducible Tregs, including *Foxp3*-positive Tregs induced in the periphery from naïve T cells, IL-10-secreting Type I Tregs, and TGFβ-secreting Th3 cells, have been shown to be able of preventing autoimmunity [25,26,27]. It has been previously reported that the depletion of Tregs causes autoimmune diseases [28,29]. In addition, Tregs are involved in several autoimmune diseases such as multiple sclerosis, type I autoimmune diabetes, inflammatory bowel diseases, and psoriasis [30]. Narrowband UVB phototherapy, which has been used for the treatment of psoriasis, can induce tolerance mediated by Tregs [31]. Suppressive function of peripheral blood Tregs from psoriasis patients was restored by UVB therapy [32]. Furthermore, retinoic acid, which has been used for psoriasis, is secreted by a particular subset of DCs in the gut-associated lymphoid tissue, inducing Foxp3-expresssing cells from naïve T cells [33]. Tregs have been shown to express TLRs [34], and endogenous TLR ligands might drive the inflammation through the modulation of Tregs and antigen-presenting cells, resulting in abrogation of suppression and inflammatory cytokine secretion [35]. TLR2/1 ligand (Pam3CSK4) is reported to induce the proliferation of Tregs [21,36]. The number of Tregs in the circulation of TLR2 KO mice was significantly reduced compared with their WT littermate controls [22], which was compatible with our results.

IL-10 inhibits excessive inflammatory immune responses [37] and exerts its anti-inflammatory function by limiting the secretion of proinflammatory cytokines, such as TNFα, Th1 cytokines, and Th17 cytokines [38,39]. Furthermore, IL-10 is needed for Tregs to sustain the expression of Foxp3. Especially, DC-derived IL-10 is important for the proper development of a subset of Tregs involved in the regulation of effector T lymphocytes [40]. The secretion of IL-10 and TGFβ by Tregs, in turn, is responsible for their effects on innate and adaptive immunity [41]. Notably, previous studies have indicated that TLR2 signaling in splenic DC induces expression of IL-10 and retinaldehyde dehydrogenase RALDH, which converts vitamin A to retinoic acid, which is critical for inducing Tregs [42,43]. Consistent with these previous studies, flow cytometric analysis of the lymph nodes revealed that the frequency of Tregs among CD4-positive cells was decreased in TLR2 KO mice. IL-10 production from Tregs and DCs was increased with either Pam2CSK4 (TLR2/1 ligand) or Pam3CSK4 (TLR2/6). Stimulation with Pam3CSK4 also enhanced the proliferation of Tregs, while that of Pam2CSK4 did not significantly increase Treg number. TLR2/1 signaling might be stronger than the TLR2/6 signaling pathway, although further study is necessary. Finally, the adoptive transfer of Tregs from WT mice ameliorated imiquimod-induced psoriasis skin inflammation and reduced *IFNγ* mRNA expression in the lesional skin, revealing the importance of Tregs in an imiquimod-induced psoriasis mouse model.

Although *TNFα* and Th1 cytokines in the lesional skin after imiquimod application were upregulated in TLR2 KO mice, Th17 cytokines except *CCL20* were unchanged despite a decrease in Tregs and *IL-10* expression. This apparent contradiction might be caused by the different TLR2 expressions on γδ T cells. IL-17-producing γδ T cells have been shown to play pivotal roles in imiquimod-induced psoriasis mouse model [44,45]. A recent report has suggested that IL-17-positive γδ T cells express TLR2, while γδ T cells producing IFNγ do not [46]. The decrease in IL-17-positive γδ T cells that might occur in the absence of TLR2 expression may be neutralized by the effect of a decrease in Tregs and IL-10 expression, leading to the unchanged Th17 cytokines in TLR2 KO mice. TLR2 has also been implicated in the production of IL-17A by Tregs [47]. In murine models, when TLR2 that is expressed on DCs is stimulated by its ligands, the DCs produce proinflammatory cytokines, and the suppressive function of Tregs is reversed. When conventional Tregs are stimulated by TLR2 ligands in the presence of DCs and Th17-inducing cytokines like IL-6, IL-23, and IL-1β, the Tregs can produce IL-17A without losing Foxp3 expression, although 20% of Tregs lose Foxp3. Considering our findings together with previous literature, we may say that TLR2 signaling in Tregs can induce both inflammatory and anti-inflammatory responses. The effect of a decrease in IL-10 expression may outweigh that of a decrease in IL-17A expression in the mouse model in this study. A decrease in IL-17A-producing Tregs, as well as IL-17A-producing γδ T cells, might account for unchanged *IL-17A* expression in the skin after imiquimod application in TLR2 KO mice.

Our results demonstrate that TLR2 plays a pivotal role in the pathophysiology of psoriasis, suggesting that enhancement of TLR2 signaling may be a novel therapeutic approach to psoriasis. TLR2 is activated by saturated fatty acids, leading to the expression of proinflammatory target genes [48]. However, rich sources of dietary saturated fatty acids, such as butter fat, meat fat, and plant oils, cannot be recommended for psoriatic patients; on the contrary, omega-3 polyunsaturated fatty acids and eicosapentaenoic acids, which downregulate TLR2 signaling pathways, are often used to treat psoriasis. This fact suggests that induction of inflammatory cytokines mediated via TLR2 signaling pathways is more important than induction of an anti-inflammatory cytokine, IL-10, produced from DCs or Tregs through TLR2 signaling pathways in humans. Therefore, modulation of TLR2 signaling in a cell-specific fashion would be a future therapeutic strategy. Although cell-specific signaling modulation may be currently difficult, Brentuximab vedotin is a good example. The agent is an anti-CD30 antibody conjugated with monomethyl auristatin E. Only CD30-expressing cells internalize the component and die by inhibition of cell division. Therefore, if we use antibody specific to activated DCs, it would be possible to block TLR2 signaling only in activated DCs without affecting TLR2 signaling in Tregs. Moreover, our findings have suggested TLR2 blocking agents, which may be developed soon, can exacerbate psoriasis by decreasing Tregs and IL-10 production.

**Figure 7:**
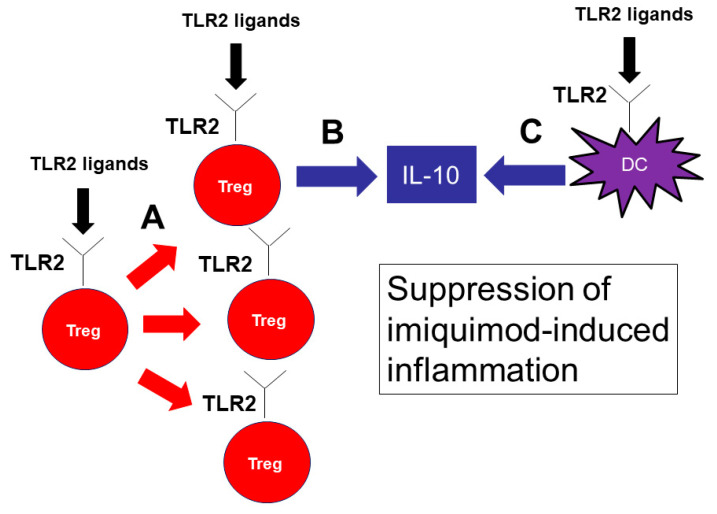
TLR2 signaling is important for the proliferation of Tregs and IL-10 production by Tregs and DCs, suppressing imiquimod-induced inflammation. TLR2 signaling is important for the proliferation of Tregs (**A**). TLR2 ligands also induce IL-10 production by Tregs (**B**) and DCs (**C**). IL-10 is the key cytokine to suppress imiquimod-induced inflammation.

In conclusion, our results demonstrate that TLR2 has an important role in the pathophysiology of psoriasis. TLR2 signaling promotes IL-10 production from DCs and Tregs and proliferation of Tregs, resulting in a decreased production of inflammatory cytokines and a marked attenuation of imiquimod-induced psoriasis skin inflammation (Figure 7). Cell-specific delivery of TLR2 signaling may be a novel therapeutic approach to psoriasis.

## 4. Materials and Methods

### 4.1. Mice

TLR2 knockout (TLR2 KO) mice were purchased from Jackson Laboratory (Bar Harbor, ME, USA). Mice were all 8–12 weeks old for all experiments. Age- and sex-matched wild-type (WT) C57BL/6 mice (CLEA Japan, Tokyo, Japan) were used as controls for TLR2 KO mice. All mice were maintained in a specific pathogen-free barrier facility. They were healthy, fertile, and did not display evidence of infection or disease. All studies and procedures were approved by the Committee on Animal Experimentation of the University of Tokyo (P17-103, 15 February 2018).

### 4.2. Induction of Psoriasiform Skin Inflammation by Imiquimod

A daily topical dose of 62.5 mg of commercially available imiquimod cream (5%) (Beselna Cream; Mochida Pharmaceuticals, Tokyo, Japan) was applied to the shaved back skin and ears of mice for 5 consecutive days (days 0–4). In some experiments, mice were intravenously transferred with PBS or Tregs from WT or TLR2 KO mice. Disease severity was assessed by using a scoring system based on the clinical psoriasis area and severity index. To be precise, erythema, scaling, and thickening were scored independently on a scale from 0 to 4 (0, none; 1, slight; 2, moderate; 3, marked; 4, very marked), and the cumulative score was used as a total score (scale 0–12). Disease severity was assessed by two researchers in a blinded manner.

### 4.3. Histological and Immunohistochemical Analysis

Samples of imiquimod-applied mouse back skin were harvested. They were formalin-fixed and stained with hematoxylin and eosin. For immunohistochemistry, mouse skin was snap-frozen and stored at –80 °C. Cryosections were fixed with cold acetone for 5 min and incubated overnight at 4 °C with anti-mouse major histocompatibility complex class II antibody (1/50 dilution, Abcam, Cambridge, UK), anti-mouse CD3 antibody (1/100 dilution, Abcam), anti-mouse Gr-1 antibody (1/100 dilution, BD Pharmingen, San Diego, CA) or anti-mouse F4/80 antibody (1/100 dilution, Bio-Rad, Hercules, CA). Tissues were subsequently stained with an avidin-biotin peroxidase complex using a Vector ABC staining kit (Vector Laboratories, Burlingame, CA, USA). Diaminobenzidine was used for visualizing the staining, and counterstaining with Mayer hematoxylin was performed according to the manufacturers’ instructions. Stained cells were counted in 10 random grids under high original magnification (×400) power fields of a light microscope. Each section was examined independently by two investigators in a blinded manner.

### 4.4. RNA Isolation and Quantitative Reverse-Transcription PCR Analysis

RNA was obtained from the back skin with RNeasy Fibrous Tissue Mini Kit (QIAGEN, Valencia, CA, USA). Complementary DNA was synthesized using Rever Tra Ace qPCR RT Master Mix (Toyobo, Osaka, Japan). The quantitative reverse-transcription PCR assay was carried out using SYBR Green PCR Master Mix (Toyobo) on an ABI Prism 7000 sequence detection system (Life Technologies, Carlsbad, CA, USA). The messenger RNA (mRNA) levels were normalized to those of the glyceraldehyde-3-phosphate dehydrogenase (*GAPDH*) gene. The relative change in the levels of genes of interest was determined by the 2^–ΔΔCT^. Specific primers for *IL-17A*, *IL-17F*, *IL-22*, and *EBI-3* were purchased from Applied Biosystems (Applied Biosystems, Foster City, CA, USA). Primers for other cytokines or molecules are shown in Table S1.

### 4.5. Flow Cytometric Analysis

Lymphocytes from draining lymph nodes of the lesional skin (i.e., axillary and inguinal lymph nodes) were obtained two days after imiquimod application. For intracellular cytokine staining experiments, cells were stimulated with 25 ng/mL of phorbol myristate acetate and 1 μg/mL of ionomycin (Sigma-Aldrich, Saint Louis, MO, USA) for 5 h, in the presence of 1 μg/mL brefeldin A (BD Pharmingen) for the last 2 h. Staining was performed according to the protocol of the anti-mouse/rat Foxp3 staining set (eBioscience, San Diego, CA, USA), using anti-CD4 (RM4–5; BioLegend, San Diego, CA, USA), anti-CD25 (PC01; BioLegend), anti-IL-10 (JES5-16E3; BioLegend) and anti-Foxp3 (FJK-16s; eBioscience) antibodies. Background staining was assessed using non-reactive isotype-matched control monoclonal antibodies (Biolegend). Cells were analyzed on a FACS Verse flow cytometer (BD Biosciences).

### 4.6. Cell Isolation and Culture

CD4^+^CD25^+^ regulatory T cells were isolated by MACS beads from axillary and inguinal lymph nodes, according to the protocol of CD4^+^CD25^+^ Regulatory T Cell Isolation Kit (Miltenyi Biotech, Bergisch Gladbach, Germany). CD11c^+^ DCs from lymph nodes were selected with anti-CD11c beads according to the protocol of CD11 MicroBeads (Miltenyi Biotech).

Cells were cultured in RPMI medium 1640 supplemented with 10% heat-inactivated FCS (Harlan)/2 mM l-glutamine/100 units/mL penicillin/100 g/mL streptomycin/0.05 M 2-mercaptoethanol (complete medium). Tregs (1.0 × 10^4^ cells per 200 μL well) were activated by plate-bound αCD3 antibody (5 μg/mL; R&D Systems) in a flat-bottom 96-well plate (Nunc), and proliferation assays were performed using BrdU Cell Proliferation ELISA Kit (Roche, Basel, Switzerland) for up to 3 days before the incorporation of BrdU in the last 8 h of culture. In some experiments, 5 μg/mL of Pam2CSK4 or Pam3CSK4 (InvivoGen, San Diego, CA, USA) were added [21]. Pam2CSK4 is a synthetic diacylated lipopeptide that induces signaling through TLR2/6. Pam3CSK4 is a synthetic triacylated lipopeptide that activates the TLR2/TLR1 heterodimer.

DCs or Tregs (2.0 × 10^5^ cells per 200 μL well) were stimulated with 100 ng/mL of Pam2CSK4 or Pam3CSK4 (InvivoGen) for 24 h [49]. Total RNA was then isolated using Trizol (Invitrogen), and mRNA expression levels were determined as mentioned above. The cell culture supernatants were measured for IL-10 by ELISA (R&D Systems, Minneapolis, MN, USA).

### 4.7. Intravenous Injection of Pam3CSK4 and Pam2CSK4

We injected into wild-type mice 50 g of Pam3CSK4 and 50 g of Pam2CSK4 or PBS intravenously two days before, on the same day and two days after imiquimod application. Messenger RNA expression in the lesional skin two days after the administration of imiquimod was examined.

### 4.8. Adoptive Transfer of Tregs

CD4^+^CD25^+^ regulatory T cells were isolated, as mentioned above. TLR2 KO mice were injected i.v. with 1.0 × 10^5^ Tregs from WT mice or TLR2 KO mice one day before imiquimod application. The number of transferred Tregs was determined in reference to similar experiments using Bregs [50]. Messenger RNA expression in the lesional skin two days after imiquimod application was further analyzed as mentioned above.

### 4.9. Statistical Analysis

Data obtained are presented as mean ± SEM. Statistical analysis was carried out with one-way ANOVA with Bonferroni post hoc tests for multiple group comparisons and the two-tailed unpaired *t*-test for two-group comparisons. For comparing two group values that did not follow Gaussian distribution, the two-tailed Mann–Whitney U-test was used. Values of *p* < 0.05 were considered to represent a significant difference.

## Supplementary Materials

Supplementary materials can be found at https://www.mdpi.com/1422-0067/21/22/8560/s1.

## Abbreviations

TLR	toll-like receptor
Treg	regulatory T cell
DC	dendritic cell
mRNA	messenger RNA
KO	knockout
WT	wild-type
