# Supplementary material for: TLR2 Deficiency Exacerbates Imiquimod-Induced Psoriasis-Like Skin Inflammation through Decrease in Regulatory T Cells and Impaired IL-10 Production
Source: Int J Mol Sci. 2020 Nov 13;21(22):8560. doi: 10.3390/ijms21228560 (PMC7696365; doi:10.3390/ijms21228560)
Supplement: Supplementary file 1 [file ijms-21-08560-s001.pdf]

Table S1 Primer sequences

Gene name (symbol)	Primer sequences
GAPDH	F: 5′-CGT GTT CCT ACC CCC AAT GT-3′
	R: 5′-TGT CAT ACT TGG CAG GTT TCT-3′
CCL20	F: 5′-TGC TCT TCC TTG CTT TGG CAT GGG TA-3′
	R: 5′-TCT GTG CAG TGA TGT GCA GGT GAA GC-3′
IL-23p19	F: 5′-TGT GCC TAG GAC TAG CAG TCC TGA-3′
	R: 5′-TTG GCG GAT CCT TTG CAA GCA GAA-3′
IL-12/23p40	F: 5′-CTC ACA TCT GCT C-3′
	R: 5′-AAT TTG GTG CTT CAC A-3′
TNFα	F: 5′-ACC CTC ACA CTC AGA TCA TCT TC-3′
	R: 5′-TGG TGG TTT GCT ACG T-3′
IL-27p28	F: 5′-GGC CAT GAG GCT GGA TCT C-3′
	R: 5′-AAC ATT TGA ATC CTG CAG CCA-3′
IFNγ	F: 5′-AGC AAC AGC AAG GCG AAA A-3′
	R: 5′-CTG GAC CTG TGG GTT GA-3′
CXCL9	F: 5′-TGG GCA TCA TCT TCC TGG AG-3′
	R: 5′-CCG GAT CTA GGC AGG TTT GA-3′
CXCL10	F: 5′-CCC ACG TGT TGA GAT CAT TG-3′
	R: 5′-CAC TGG GTA AAG GGG AGT GA-3′
Foxp3	F: 5′-TAC TTC AAG TTC CAC AAC ATG CGA CC-3′
	R: 5′-CGC ACA AAG CAC TTG TGC AGA CTC AG-3′
IL-10	F: 5′-TTT GAA TTC CCT GGG TGA GAA-3′
	R: 5′-ACA GGG GAG AAA TCG ATG ACA-3′
TGFβ	F: 5′-TTG CTT CAG CTC CAC AGA GA-3′
	R: 5′-TGG TTG TAG AGG GCA AGG AC-3′

F and R primers used for gene expression analyses of the indicated genes.

F, forward; R, reverse.
